# Management of an Open Apex Using a Platelet-Rich Fibrin Membrane as an Internal Matrix and Mineral Trioxide Aggregate as an Apical Barrier: A Case Report

**DOI:** 10.7759/cureus.75316

**Published:** 2024-12-08

**Authors:** Ruchali S Bhandare, Sudha Mattigatti

**Affiliations:** 1 Department of Conservative Dentistry and Endodontics, School of Dental Science, Krishna Vishwa Vidyapeeth (Deemed To Be University), Karad, IND

**Keywords:** apexification, apical barrier, chronic apical periodontitis, mta, non-vital tooth, prf, pulpal necrosis

## Abstract

Apexification is a crucial procedure for achieving apical healing in non-vital teeth with open apices. Traditionally, calcium hydroxide has been used for this purpose, but it has significant drawbacks, including prolonged treatment duration, increased risk of root fracture, and the potential for porous barrier formation. Mineral trioxide aggregate (MTA) has emerged as a superior alternative due to its biocompatibility, faster setting time, and better sealing properties. However, MTA extrusion into periradicular tissues can cause persistent discomfort. The use of an internal matrix such as platelet-rich fibrin (PRF) can help contain MTA within the root canal, enhancing treatment outcomes.

A 35-year-old female presented with dull, aching pain in tooth 21, a history of trauma 10 years prior, and incomplete root canal therapy 2 years ago. Diagnosis revealed an open apex with chronic apical periodontitis and pulpal necrosis. Initial management involved canal disinfection with sodium hypochlorite (NaOCl) and calcium hydroxide as intracanal medication. One week later, the patient returned asymptomatic. PRF was prepared from the patient's blood and used as an internal matrix. Mineral trioxide aggregate was then placed against the PRF membrane to form an apical stop. The canal was subsequently obturated with thermoplasticized gutta-percha and restored with composite resin.

The use of PRF and MTA provided effective apical sealing, preventing material extrusion and promoting tissue healing. The PRF matrix facilitated the controlled placement of MTA, minimizing complications and enhancing periapical healing. The combination of PRF as an internal matrix and MTA for apical barrier formation represents a promising approach for managing non-vital teeth with open apices. This technique ensures better control over material placement, reduces treatment time, and improves the overall success of endodontic therapy. The aim of this case report is to describe the endodontic management of a non-vital permanent tooth with an open apex and chronic apical periodontitis using a single-step apical barrier technique with MTA and PRF as an internal matrix.

## Introduction

In order to achieve apical healing, apexification is an endodontic procedure in which the apex of an exposed tooth is covered with a calcific barrier. Usually, non-vital pulp in permanent teeth with an open apex is treated with this treatment. For endodontic treatment to be effective over the long term, the root canal system must be completely cleaned, shaped, and then homogeneously obturated. There are instances where the natural apical constriction is problematic, such as during the growth of teeth. Making a barrier or apical stop permits the application of root canal filling material in opposition to it without going overboard with extrusion, which is one of the objectives of endodontic therapy [1].

An apical seal is the primary objective of treatment for teeth with pulpal necrosis. This apical seal was previously accomplished by generating hard tissue to act as a barrier. The primary drawbacks of the calcium hydroxide apexification process are the root's thin walls, which are prone to fracturing, and the barrier's porous nature, which may include some soft tissue even if it is calcified [2].

Several materials have been suggested in the literature as an alternative to the conventional method of apexification that uses calcium hydroxide. Mineral trioxide aggregate (MTA) is the most widely used material for this process. Fine hydrophilic particles of silicate oxide (SiO), tricalcium silicate (Ca₃S), and tricalcium oxide (Ca₃O) make up MTA. It creates a colloidal gel when combined with sterile water, and it takes three to four hours to set in the presence of moisture [3]. Mineral trioxide aggregate is more biocompatible, has a pH of 12.5, less leakage, superior antibacterial qualities, high marginal adaptation, and a brief setting time (four hours) [4, 5].

The results of three occurrences of inadvertent MTA extrusion during apical barrier treatment into periradicular tissue were reported in a case study by Nosrat A, et al. (2012). They concluded that hardening of the extruded MTA may not occur and could be linked to ongoing periapical discomfort [6]. Hence, it has been suggested to use the "internal matrix concept" to limit MTA within the root canal. Dohan DM, et al. originally reported platelet-rich fibrin (PRF) in France. Platelet-rich fibrin is a member of the emerging class of platelet concentrates, which has demonstrated several benefits, including simplicity of preparation, the absence of blood's biochemical processing, which renders the preparation purely analogous, and the ability to promote the growth of bone, healing of wounds, hemostasis, and bone maturation [7]. The treatment with a single-step apical barrier placement of an immature tooth (open apex) is described in this case study. As an internal matrix, MTA and PRF membrane, which is autologous [8].

## Case presentation

The patient

A 35-year-old female patient from Dhebewadi, Karad, presented to the Department of Conservative Dentistry and Endodontics at Krishna Vishwa Vidyapeeth, Karad, complaining of a dull, agonizing pain that had persisted for three weeks. History of trauma 10 years ago, as well as incomplete root canal therapy with tooth 21, two years ago. The patient's medical history was not relevant. An intraoral examination of her teeth showed that she had tooth discoloration with respect to tooth 21. The percussion-sensitive tooth was number 21. The tooth 21's periodontal probing depth fell within the usual range. An open apex and immature root linked to periapical radiolucency were discovered on the intraoral periapical radiograph of tooth number 21.

Diagnosis

**Figure 1:**
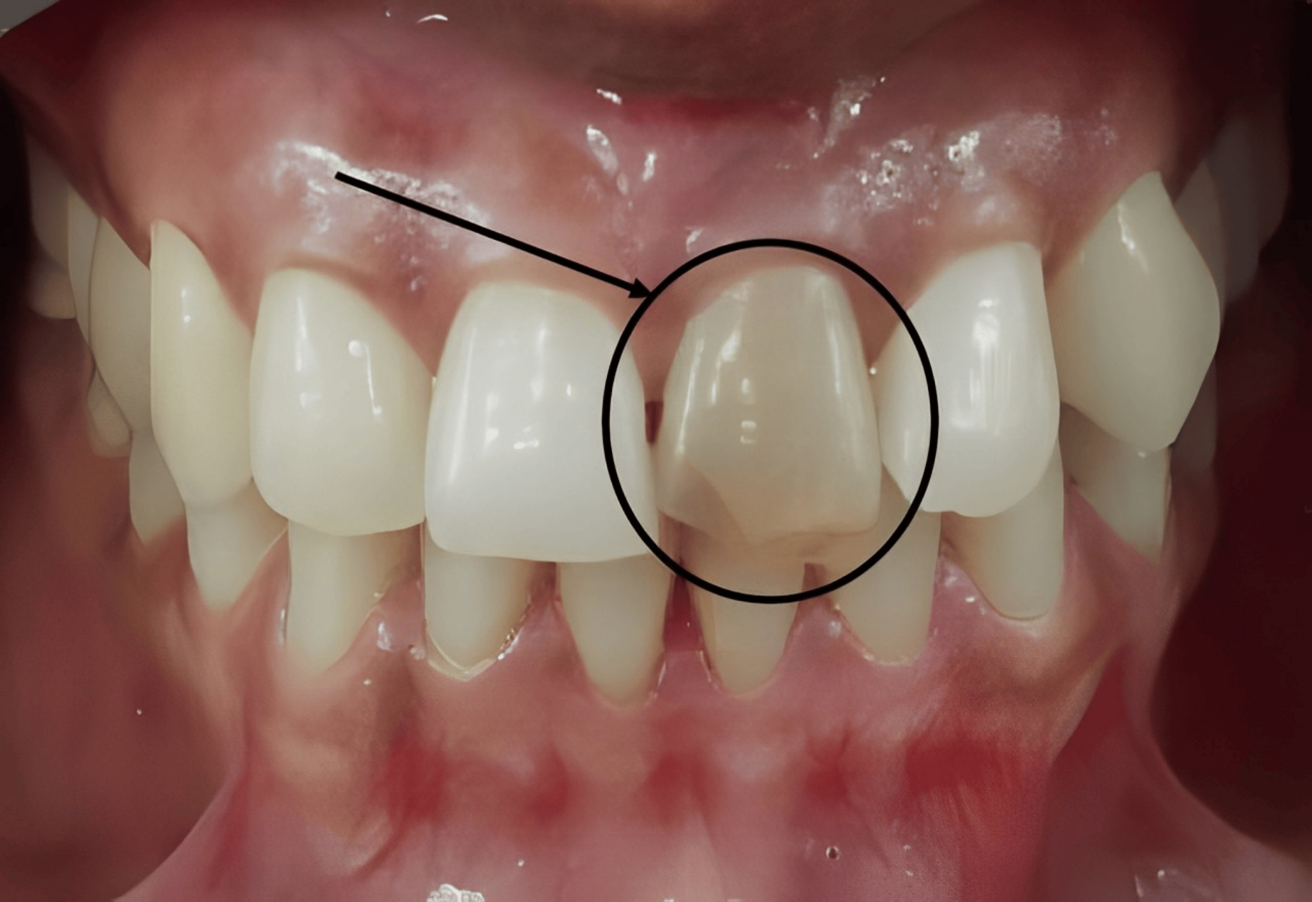
Intraoral image highlighting the infected tooth

**Figure 2:**
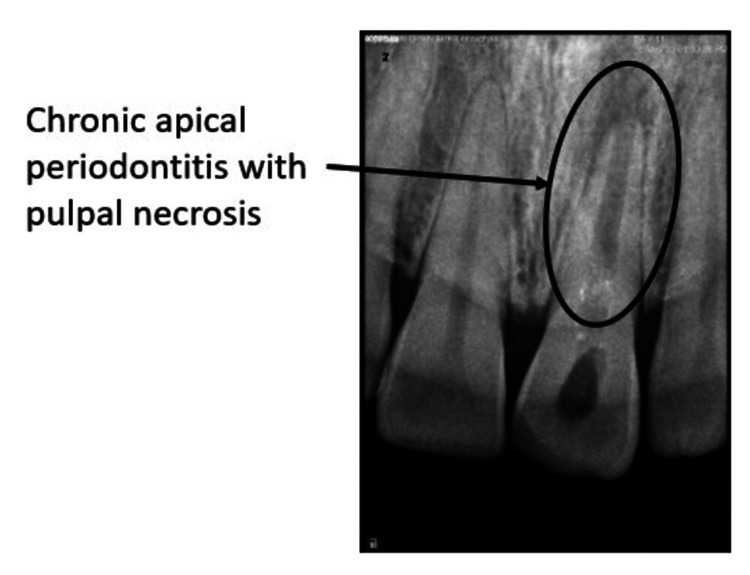
Pre-operative radiovisiography (RVG)

The clinical image, which shows a discolored tooth, before starting any procedure, is represented in Figure 1. Before performing any procedures, a radiograph was taken, which helped in diagnosing an open apex with chronic apical periodontitis and pulpal necrosis. It can be seen in Figure 2.

Management

**Figure 3:**
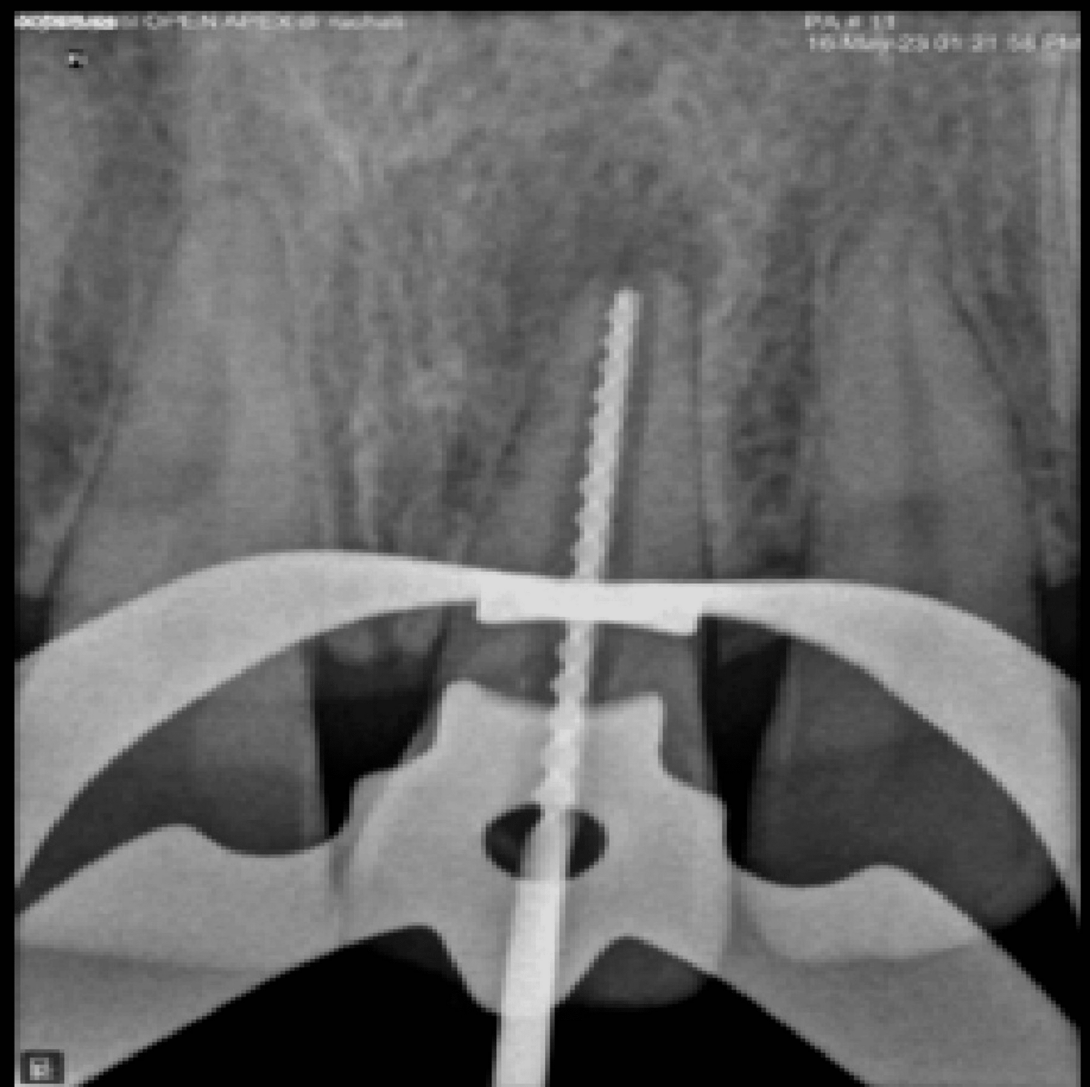
Working length radiovisiography

**Figure 4:**
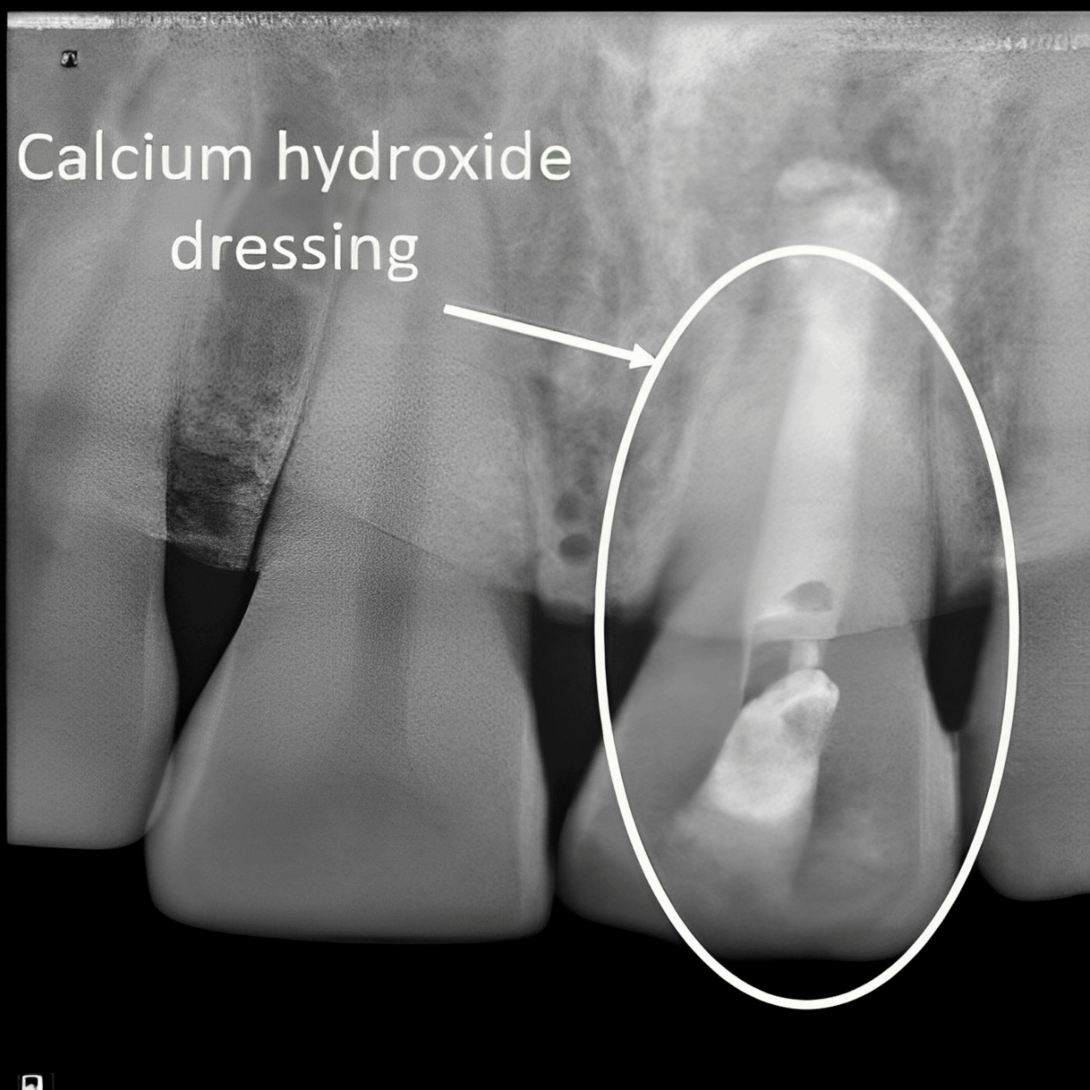
Calcium hydroxide dressing

Firstly, the rubber dam was put in place. The working length was ascertained, as shown in Figure 3, and the endodontic access cavity was re-accessed. With K-files (Mani Inc., Tochigi, Japan), the canal was lightly instrumented. It was then irrigated thoroughly with sodium hypochlorite (NaOCl) (1%), absorber paper points (Dentsply Sirona, Charlotte, NC, USA), and saline (0.9%). Calcium hydroxide (Ca(OH)₂) was then added for intracanal medication and temporary securing access with Cavit G (3M ESPE, Bayern, Germany). A radiograph of calcium hydroxide dressing is shown in Figure 4. After a week, the patient was summoned back. The tooth was asymptomatic after a week.

During this appointment, it was decided to install MTA (Angelou's, Dentsply Sirona) as an apical stop and employ the membrane of PRF as an internal matrix. The patient's written informed permission was acquired following a comprehensive explanation of the clinical procedure and associated risks. Sodium hypochlorite was utilized for the irrigation of canals, plus absorbent paper points were used to dry them (Dentsply Sirona).

Preparation of the PRF membrane

**Figure 5:**
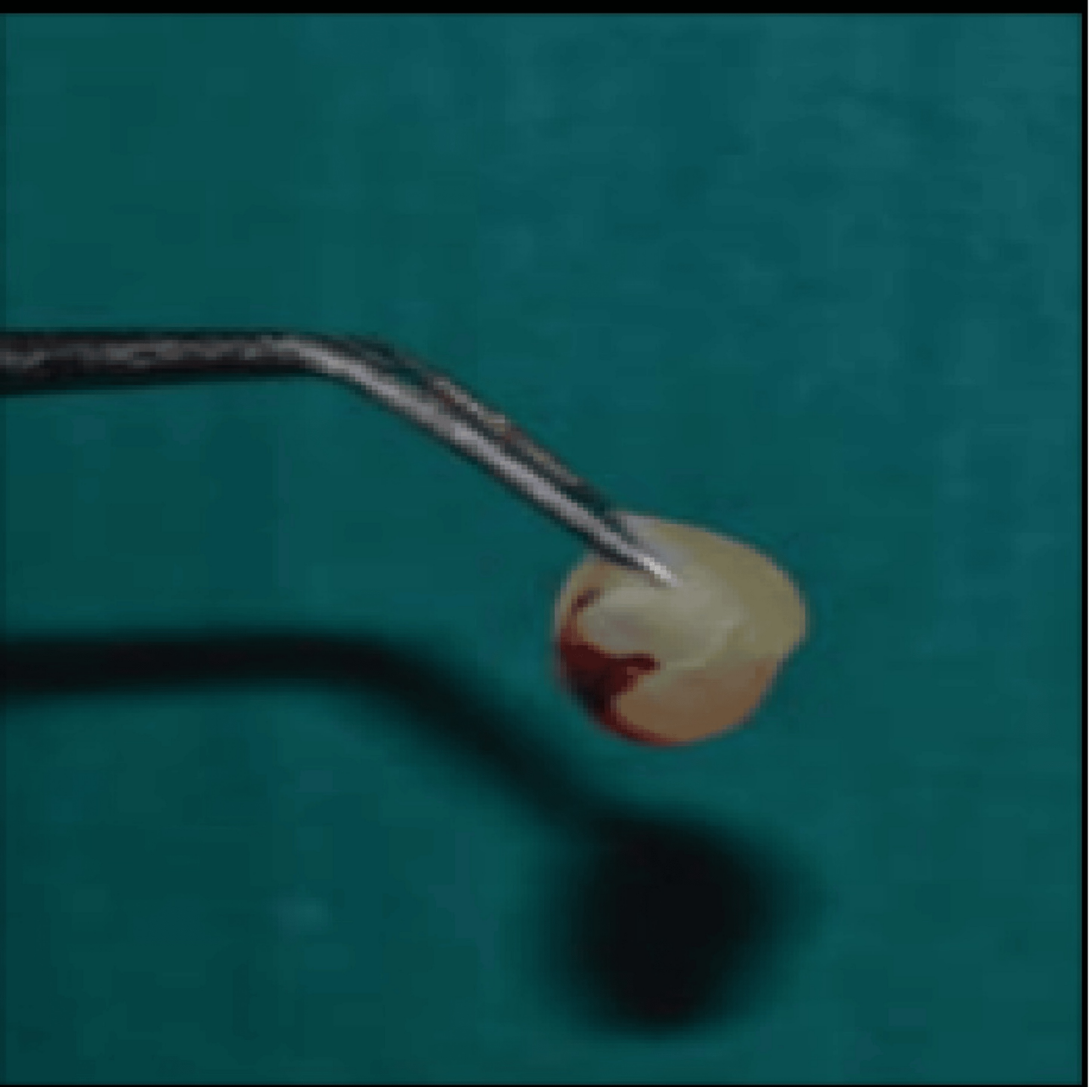
Platelet-rich fibrin membrane

**Figure 6:**
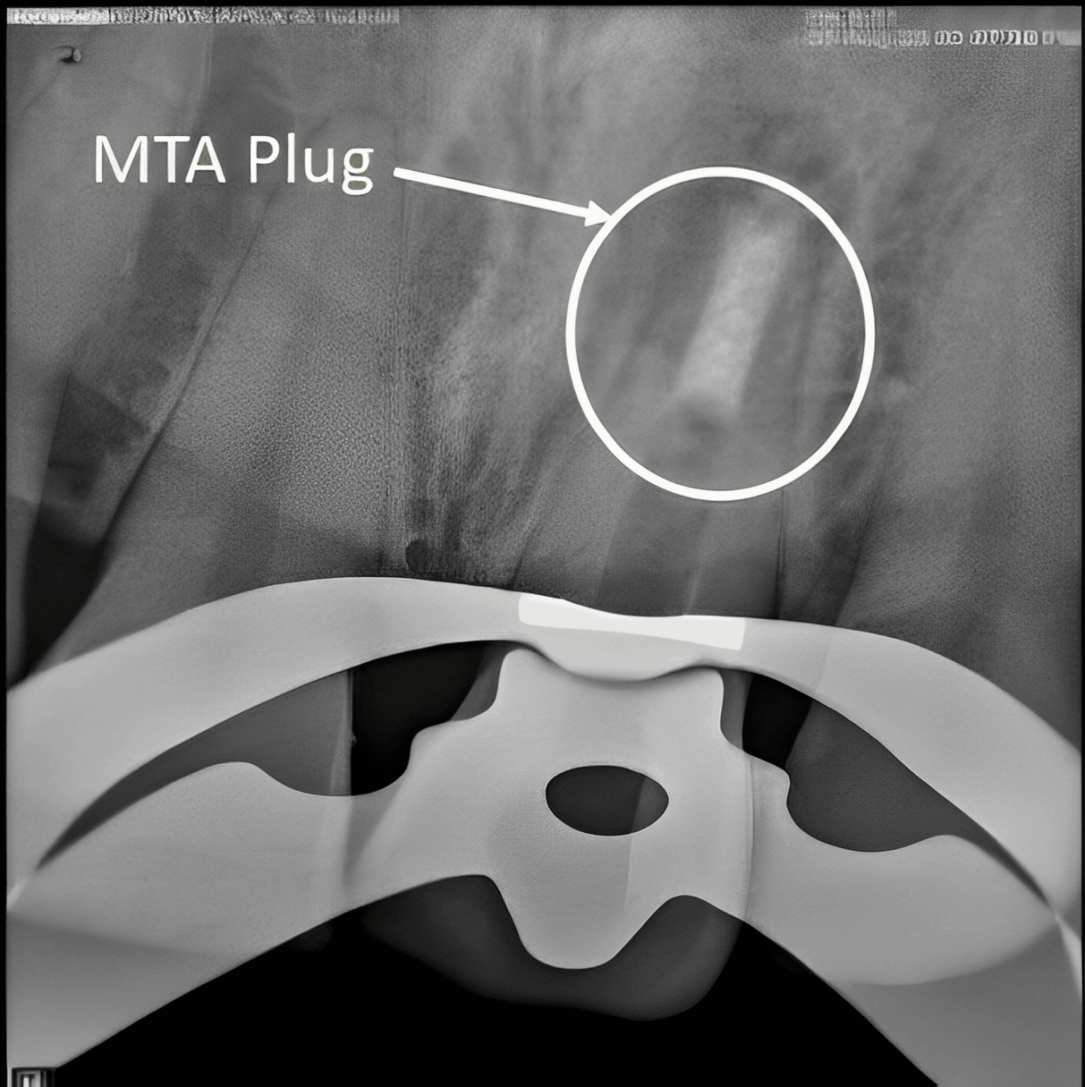
Mineral trioxide aggregate (MTA) plug given

Thirty minutes before the clinical procedure, the antecubital vein’s venipuncture was used to remove 10 milliliters of whole blood. After collecting blood in a 10-mm sterile glass tube having no anticoagulant, the tube was spun for 10 minutes at a speed of 3000 revolutions per minute. Three layers make up the final product: at the top, an acellular platelet-poor plasma; in the middle, a PRF clot; and at the bottom, red blood cells. To obtain a PRF membrane, the PRF clot was removed, and then its contents were pressed out. With an operating microscope, the membrane (PRF) was carefully compacted with hand pluggers to create a barrier or stop at the apex. The PRF membrane is shown in Figure 5. After being inserted into the canals, MTA was crushed with pluggers up in opposition to the PRF membrane. After that, a radiograph was obtained. The MTA is positioned appropriately to generate an apical stop that is roughly 3-4 mm thick, as shown in Figure 6.

**Figure 7:**
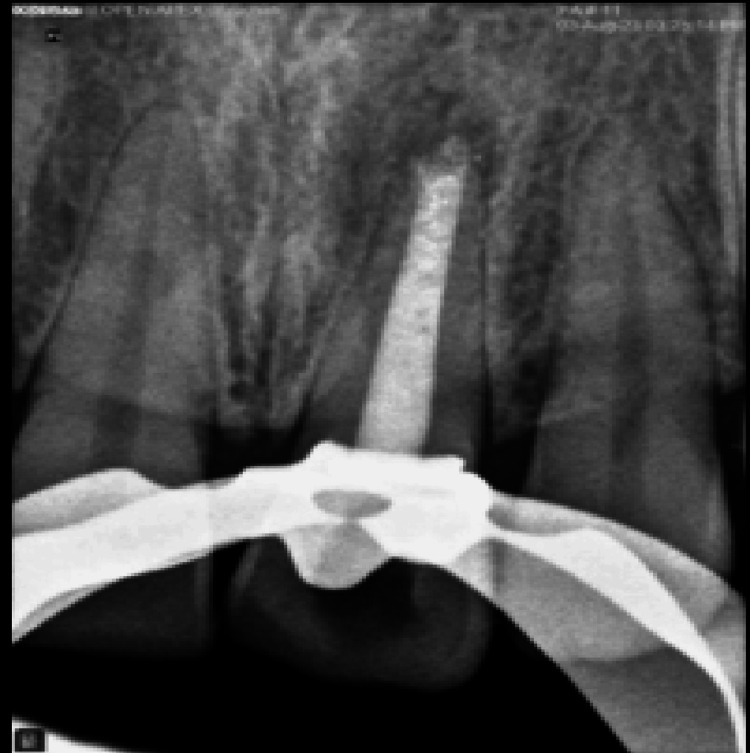
Obturation done with thermoplastized technique

**Figure 8:**
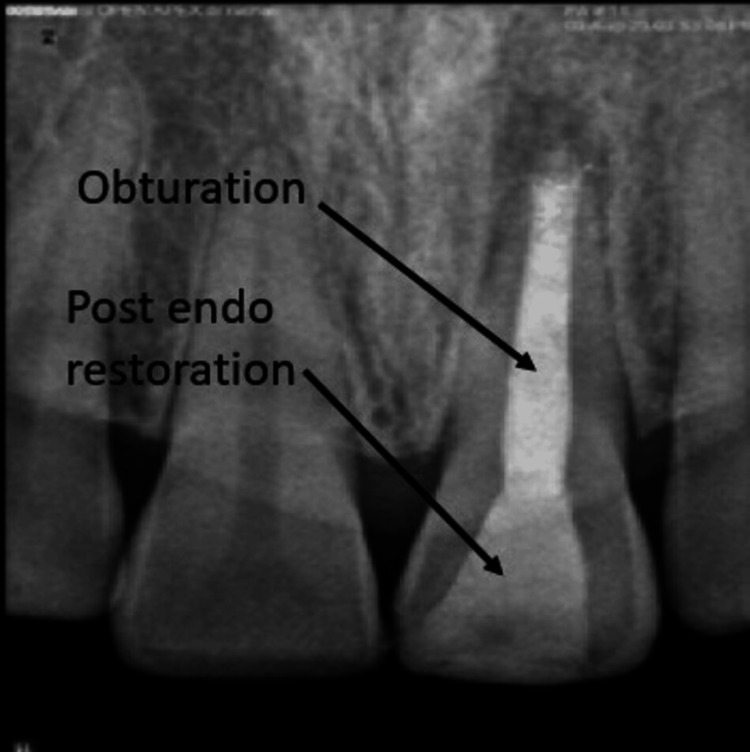
Post obturation and problem-oriented record radiovisiography

To encourage setting, the blunt end of a large paper point was wet with water and placed in the canal. Intermediate restorative material (IRM), a temporary filling material, was used for sealing the access cavity. The patient was called back the next day, and isolation of the tooth was performed. To ensure a rigid set, a hand plugger was gently pushed on the MTA plug. The rest of the canal was then sealed using thermoplasticized gutta-percha as shown in Figure 7, and composite restoration was completed as a post-obturating procedure, which is shown in Figure 8.

Patient follow-up

**Figure 9:**
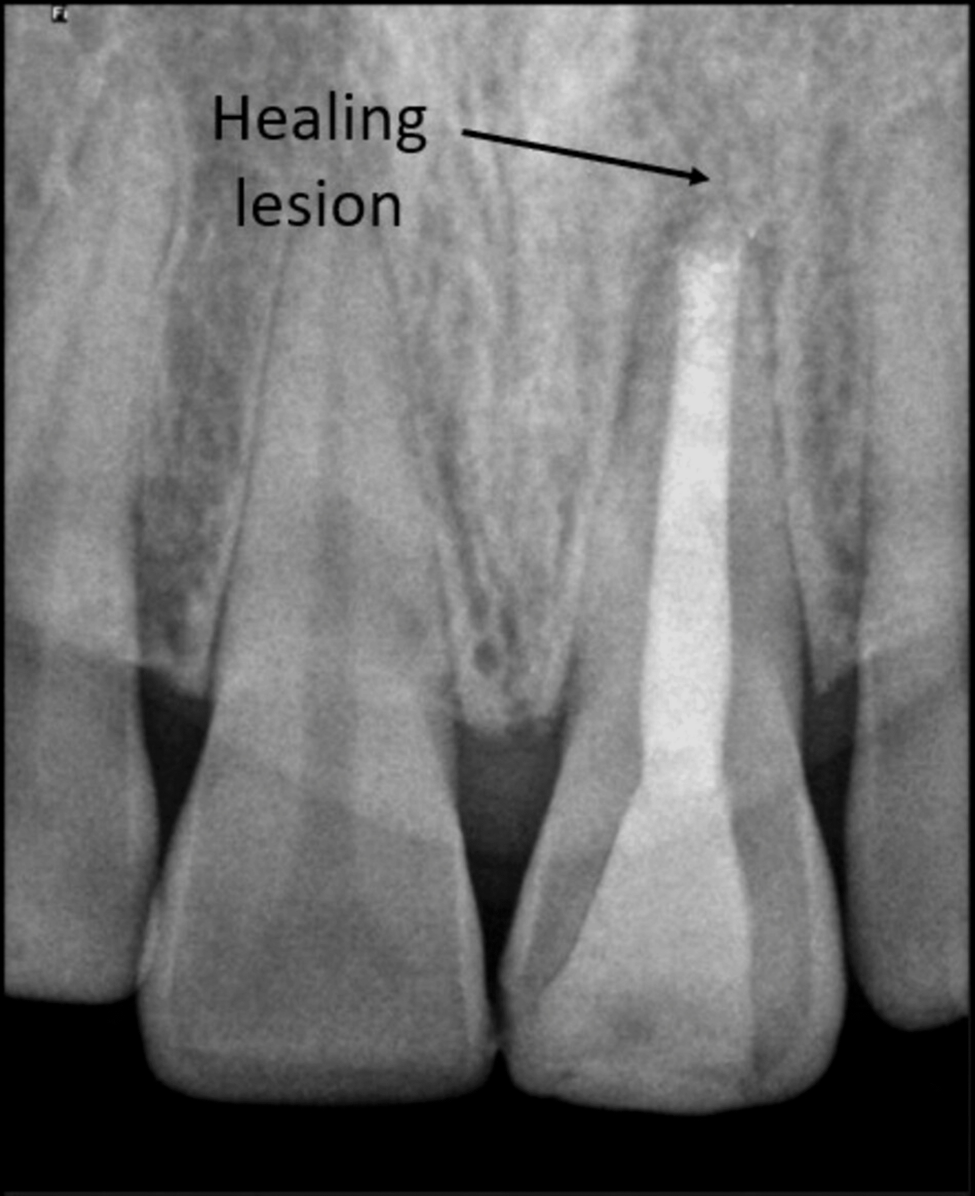
Follow-up radiograph

The patient was recalled for follow-up examinations to check for clinical and radiographic signs of healing at time intervals of three months. On clinical examination, there were no signs such as tenderness on percussion, and there was no presence of a sinus tract. On radiographic examination, it showed a healing periapical lesion as shown in Figure 9.

## Discussion

The patient had a history of trauma from 10 years back. The trauma resulted in pulpal necrosis, which led to discoloration of teeth along with periodontal tissue injury and open apex formation. Which can also occur in intrusion, luxation, or avulsion, leading to external resorption. A blunted or shorter root with an open apex may be the consequence of early pulp necrosis, inadequate root development, or external resorption of the root as a result of trauma [9,10].

The main issue seen in the patient with an open apex is the extrusion of material into the apical region and inadequate sealing of the apex of the tooth. Excessive amounts of extruded material can cause an inflammatory reaction that persists, potentially complicating or even preventing tissue repair [6]. By using a matrix, one can prevent material from being extruded inside the periodontal tissue, minimize leakage, and promote the repair of periodontal tissues [11]. Various materials have been used for barrier formation, which includes calcium hydroxide, hydroxyapatite, resorbable collagen, and PRF. Many growth factors, such as vascular endothelial growth factor, platelet-derived growth factor, and transforming growth factor B, are found in platelets. Platelet-rich fibrin is an immunological platelet concentration; it promotes immunity and healing when it is gathered on a single fibrin membrane. Its low thrombin-content molecular structure serves as the ideal matrix for fibroblast and endothelial cell migration. It enables simpler fibrin remodeling and quicker angiogenesis. Thus, it possesses all the necessary elements for the best possible healing [12-14].

By using a matrix, material is prevented from extruding into periodontal tissues, along with proper sealing of material and positive tissue response [15]. During specification, a range of materials have been utilized in the formation of the apical barrier. Calcium hydroxide has been the preferred material for creating the apical calcific barrier [16]. Nevertheless, the duration required to cause apical end closure limits the use of Ca(OH)₂. Furthermore, the wall of the root canal becomes more fragile when calcium hydroxide is used for a prolonged period. Mineral trioxide aggregate could be a useful stand-in to do this task with reliable results. Proper peri-radicular architecture can be maintained by MTA by creating a hard-tissue barrier [17-19]. Mineral trioxide aggregate is an excellent material for apexification because of its better biocompatibility, ability to harden in the presence of blood, and possibility for quick tooth repair and not compromise the mechanical properties of dentine [20-23].

The primary concern in nonvital tooth treatment is the removal of microorganisms from the root canal system. Since you cannot use instruments correctly in teeth with open apices, calcium hydroxide is utilized as an intracanal dressing, and sodium hydroxide (1%) is utilized as an irrigant in lesser concentrations to clean and disinfect the root canal [24,25].

Limitations

This study has a limitation that should be acknowledged: the CBCT image can be used for detecting the presence of an open apex as it provides a three-dimensional evaluation of the apex. However, a radiovisiography revealed the presence of an open apex in the tooth of concern, so CBCT imaging was not done.

## Conclusions

The described case highlights the effective application of a single-step apexification technique using MTA in combination with a PRF membrane. The procedure provided a reliable and biocompatible apical barrier for the management of an immature tooth with an open apex. The use of PRF as an internal matrix not only prevented material extrusion but also promoted periodontal tissue healing by leveraging its autologous growth factors, white blood cells, platelets, fibrin, and stem cells, which enhances the overall treatment outcome. Mineral trioxide aggregate, which is used, is biocompatible and induces hard tissue formation. The case underscores the importance of precise clinical techniques, appropriate material selection, and interdisciplinary approaches in the successful management of complex endodontic scenarios.

Further research can be carried out as there is growing interest in regenerative procedures by using biologically based materials such as tricalcium phosphate, osteogenic protein, and bone growth factor, which can be used for apex closure in cases of open apex. This regenerative procedure with the help of stem cells, scaffolds, and growth factors, if done appropriately, has the potential to produce promising results.
